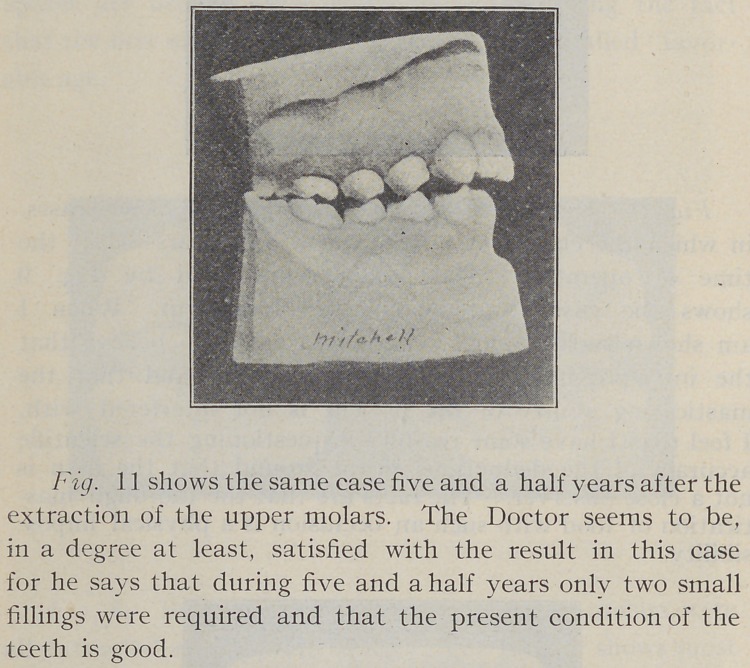# First Molar Extraction during Childhood, and Its Relation to Occlusion and Physiognomy

**Published:** 1903-11-15

**Authors:** Milton T. Watson


### FIRST MOLAR EXTRACTION DURING CHILDHOOD, AND ITS RELATION TO OCCLUSION AND PHYSIOGNOMY.

BY MILTON T. WATSON, D.D.S.

Read before the Michigan Dental Association, July 7, 1903.

Were this subject one of less importance, and were it not for the fact that it is one greatly misunderstood, I should feel some embarrassment in presenting it for your consideration, as it is one that I have spoken upon whenever opportunity has presented itself for me to utter a protest against it. I believe it to be the real curse of dentistry the world over. I also wish it to be understood that there has been no extra effort made to find new reasons for opposing the extraction of first permanent molars, for the reasons which have long been known are more than sufficient to convince any thoughtful student or close observer that the operation is always attended with evil results. I shall quote freely from current literature, as well as from former papers of my own.

A large number of the slides which I shall use to substantiate my position are shown through the courtesy of Dr. C. M. Briggs, of the University of Michigan, who prepared them with great care, and for the sole purpose of showing what actually happens when first molars are extracted. *They are shown here for the first time and an examination of the models from which these slides were made will absolutely convince any man, who is broad enough to be open to conviction, that the operation should be forever stamped as malpractice, except it be performed under conditions which would justify an amputation, were the organ under consideration a limb instead of a tooth. That these organs

* Only a limited number of the slides used in connection with this paper are here shown.—EDITOR.

may be lost among the submerged classes is to be expected, but this can never excuse a man for advocating extractions among that class which comes to him believing in his greater ability to decide such matters for their best interests.

I have only the kindliest feelings for those distinguished gentlemen who extracted first permanent molars at a time when laymen had not yet learned the need of dental attention for their young children, and when the treatment of exposed pulps and especially abscessed teeth were things little understood, and usually followed by disastrous results. These difficulties have very largely disappeared at this time—in fact almost completely so among the so-called "better class," for they know and appreciate the value of the dental apparatus—and it is our solemn duty to see to it that the laymen who have not yet reached this stage of refinement are given true light upon the subject when such light is sought, for who knows but that it shall be from among these that great singers and orators of the future may come.

The arguments in an article by Dr. W. Mitchell, of London, (*Items of Interest*, Vol. 21), are a fair type of those advanced by the men who advocate this practice. He says * * *' 'The points I wish to bring out for your consideration are why and when shall the extraction of the first permanent molars be performed?" Continuing, he says, "I propose by my deductions, based upon experience, to convince those who have heretofore been opposed to the extraction of these teeth that we have a practical and legitimate means of preventing, to a great extent, the ravages of dental caries, especially that form produced by lateral pressure, and the securing of a more serviceable dental armament by the more perfect safeguarding of the interproximal space than is possible by flat and imperfectly-contoured fillings; and later by affording patients a more perfect masticating surface; and last, but not by any means least, the satisfaction of securing to patients in the most practical way probable immunity from constant and prolonged dental operations during the greater part of their lives."

On the supposition that such practice is based upon false philosophy, (that it really is, I shall later attempt to prove), it is our duty to counteract these teachings so far as possible, for it is a truth too well known to need elaborating upon that dentists who are not surrounded by an encouraging professional environment, and who are not thus stimulated to do their very best by the fear of being outclassed by their colleagues, fall easy victims to "these easy methods of practice," and excuse themselves with the thought that certain prominent dentists of whom they have read do the same thing.

In answer to Dr. Mitchell's question, "Why and when the first permanent molars should be extracted?" I would return the flat and definite answer, NEVER, when it is possible to save them, and it is assumed that teeth with exposed pulps or even abscessed roots would not be looked upon as worthless unless every possible effort to save them had been attended with failure.

If it were true that, "the extraction of these teeth is a practical and legitimate means of preventing to a great extent, the ravages of dental caries * * *," then the man given to ridicule might be excused for carrying this same "philosophy" a little farther and advocating the removal of all the teeth and thus absolutely prevent caries, malocclusion, toothache, facial neuralgia, pyorrhea, abscesses of dental origin on the face and neck and the scars resulting therefrom; in fact, the complete eradication of all the unpleasant things that ever happen to us as a result of trouble with our natural dental organs. But, seriously, at the present time none of us need to suffer any great hardship because of caries associated with "lateral pressure and flat surfaces," for if it is a case where occlusion even approximates the normal, there will be comparatively little difficulty in securing sufficient separation to enable a skillful operator to properly contour the surfaces, and if the case is one where this is not possible, then it is evident that the mal-arrangement of the teeth is sufficiently bad to demand that they be straightened. As to this operation safeguarding the interproximal spaces, no greater blunder was ever made. I tell you nothing can guard that more surely and completely than a proper arrangement of the teeth, which, as you all well know, allows a perfect union of the gum between the teeth from the lingual and buccal sides, and yet the contact of the teeth above it is such that the heavy pressure of mastication fails to crowd particles of food between the teeth. The guarding of these spaces is unquestionably one of the most important things, so far as the future comfort of the patient is concerned, and it can only be successfully accomplished by the practically normal arrangement of the normal number of teeth, some possible exceptions being cases where third molars can be sacrificed without apparent injury.

If this is an operation of such lasting benefit, and if it is true that it does not influence the development of the face and the jaws, will some one please tell me why it is that the men who advocate first molar extraction always insist upon the removal of *all four of these teeth*? The real truth is that the development of the jaws *is* involved. If only the lower molars are taken out, a most perceptible shortening of the lower jaw will result and the effect will be noticeable not only in the occlusion but also in the facial lines. On the other hand, where only the upper molars are removed, the lack of development is quite as perceptible as in the case of the lower jaw, in some instances even allowing the upper incisors to close in lingual relation to the lowers. The cases where these results do not follow to a greater or less degree are where the cusps of the teeth are well defined and the occlusion sufficiently accurate so that the occlusal contact will carry forward the teeth of both jaws in harmony as development takes place and in these cases we often find that the space where the molar was extracted does not close up completely. With the above facts in mind, and their indisputable accuracy always borne out by a careful examination of models, I fail utterly to see how any sane man, who professes even the average amount of ability to observe closely, can stand up and attempt to make us believe that this operation does not influence the development of the jaws, and no one would have the affrontery to say that the facial development is not always envolved if the jaws are really under-developed. With the very apparent interference with the development of the jaw in cases where the extraction is confined to either the uppers or the lowers, it must be perfectly clear to any thinking man that the so-called "better results from the harmonious extraction" of these teeth is due solely to the fact that there is an equal lack of development in both jaws and not to the fact, as Mitchell and others maintain, that there is no interference with their development.

The greatest evil to the greatest number, as a result of this operation is perhaps the impaired masticating ability; this is due not only to faulty occlusion but also to the fact that this much-talked-about "inter-proximal space" is not properly safeguarded, as I will convince you later on when the projections are thrown upon the screen. I have seen these cases after extraction where, because of the lingual inclination of the remaining teeth, no lateral motion of the jaw was possible. the victim being compelled to "hobble" through the act of mastication by a simple up-and-down movement of the jaw.

The diminution in the size of the jaws as a result of these extractions may, and in certain cases does, work grave injury to the individual even to the extent of barring him from certain vocations for which he was, by nature, fitted. To begin with, the facial appearance must certainly be involved. Not a few are the times that I have seen young women with that peculiar deficient development in the region of the mouth, caused by these abominable extractions, who would otherwise have been types of striking beauty; and these same individuals were handicapped in their speech because of the lack of room for a free and normal use of the tongue. Think, gentlemen, how easily you can destroy the chances for a famous stage career. These conditions which interfere with the voice of the singer or actress are equally disastrous to the man who is by nature fitted for the lecture platform, the pulpit or the law. I happen to know, personally, a clergyman who is conceded to be one of the most profound thinkers and earnest workers in the great church which he represents, who is so handicapped for want of room in his oral cavity, and this want of room is associated with the loss of first molars and some other teeth as well, that his pulpit utterances lose greatly in their force, and he suffers keenly from an ever-present knowledge of his weakness. Are not such cases sufficient to cause us to stop and think seriously before we perform these operations?

The best result I have ever seen, where the first molars have been sacrificed, is that of a young man now some twenty years of age, and yet even in this case his grinding capacity is very greatly lessened notwithstanding the fact that the spaces are well closed, but what is even more noticeable is the fact that his speech is decidedly indistinct. The teeth are also showing very decided wear. The first molars have a most important bearing in determining the "length of the bite," and if the normal arrangement here is broken up and the jaws come closer together than nature intended, the oral space is of course lessened as a result and the lines of the lips, from a purely esthetic standpoint, must be marred, and in these cases the lower incisors are not infrequently seen to strike against the gum along the linguo-gingival line of the upper incisors, and while they have been working their way up to this point they have often, at the same time, forced the upper incisors forward at such an inclination as to make them appear very unsightly, oftimes spaces appearing between them.

There is another point to which I want to call your attention and to which I find no reference in the literature. We all know that when normal function is interfered with that the evil results may be very far reaching, and while actual dissections have not as yet been performed to substantiate what I am about to say, it is, nevertheless, not at all unreasonable to believe that when we so effectually interfere with the development of the jaws and face by the extraction of these teeth, that we may at the same time interfere with the development of the maxilla proper, and through its influence with the development of the nasal passages and consequently with normal respiration; and, if this be true, debilitated constitutions and even fatalities may occur from infections which result from mouth-breathing and which might not have occurred had the inspired air passed through the filtering process to which it would have been subjected had it been inhaled through a normal nose instead of the mouth. In cold print this danger may seem a little far fetched, but to bring the matter nearer home, who among you stands over a patient suffering from a contagious disease without closing his lips tightly for the purpose of compelling nasal respiration? Why do you do it? Some, because of their knowledge of the protection the nose thus affords; others because of the promptings of nature, of which he may be unconscious—instinct, so called.

There may be those among you who will not be willing to believe that the above-named conditions are really a source of danger to patients, but whether you are ready to accept that or not, you cannot gainsay that where nasal trouble already exists, and where the jaws are sufficiently inharmonious in their mesio-distal relations to make it necessary for the patient to put forth a subconscious effort in order to close the lips, that the loss of the lower molars, or even one of them, yes, even a bicuspid, would certainly put the patient in condition where mouth-breathing would become permanently established.

There are still many things that might be said in condemnation of the extraction of these teeth, things which are more apparent to the man viewing the situation from a purely dental standpoint, but these are apparent to any observer, and will be dwelt upon momentarily as we go over the pictures together.

*Fig.* 2. Boy nineteen years old: Lower molars extracted at thirteen years of age, resulting in an almost complete destruction of the occlusion, and showing a not uncommon result of the extraction of first permanent molars after the jaws have developed for the accommodation of the second molars.

*Fig.* 3. Man twenty-four years old: Upper molars extracted at twelve years of age. The cusps of these teeth are short, hence there is a short over-bite, and this allowed the incisors as well as the bicuspids to drift backward. The result is a greatly-impaired masticating apparatus as well as a marked facial deformity.

*Fig.* 4. Lower molars extracted at twelve years of age. The lower jaw failed to develop in length, along with the upper, the result, of course, being a receding lower jaw and a pronounced facial deformity, to say nothing of the interference with the masticating capacity of the individual.

*Fig.* 7. Girl, age twenty-three: Upper and lower first molars all extracted at twelve years of age. It is evident that at this age the second molars had advanced sufficiently to have caused considerable development of the jaws, hence the scattering of the teeth after the removal of the first molars, destroying in a great measure their usefulness as well as their appearance. The inter-proximal spaces are utterly unprotected, notwithstanding the fact that the first molars were all extracted at a so-called "favorable age."

*Fig.* 10. Shows another of Dr. Mitchell's cases at the age of about ten and a half years, at which time the upper first molars were extracted. Some months of time elapsed before the lower molars were extracted, and on account of this delay the occlusion has been modified as compared with the other case, so the Doctor tells us.

## Figures and Tables

**Fig. 1. f1:**
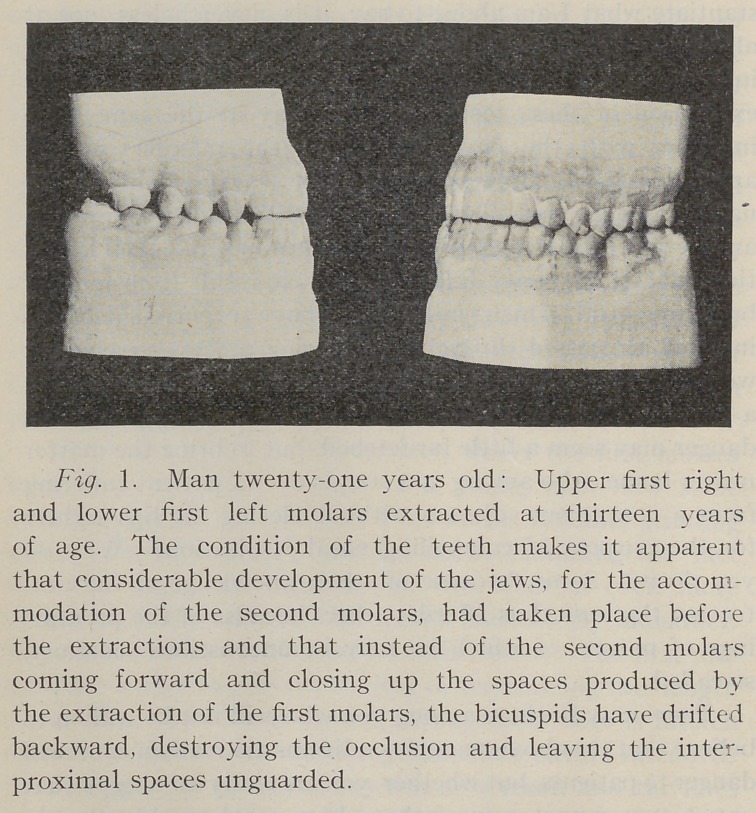


**Fig. 2. f2:**
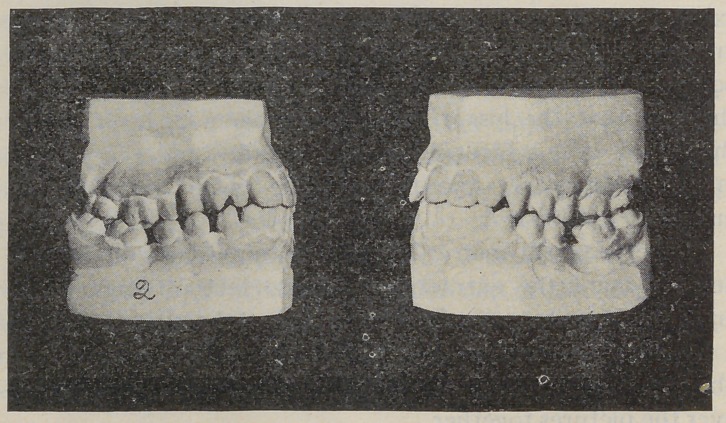


**Fig. 3. f3:**
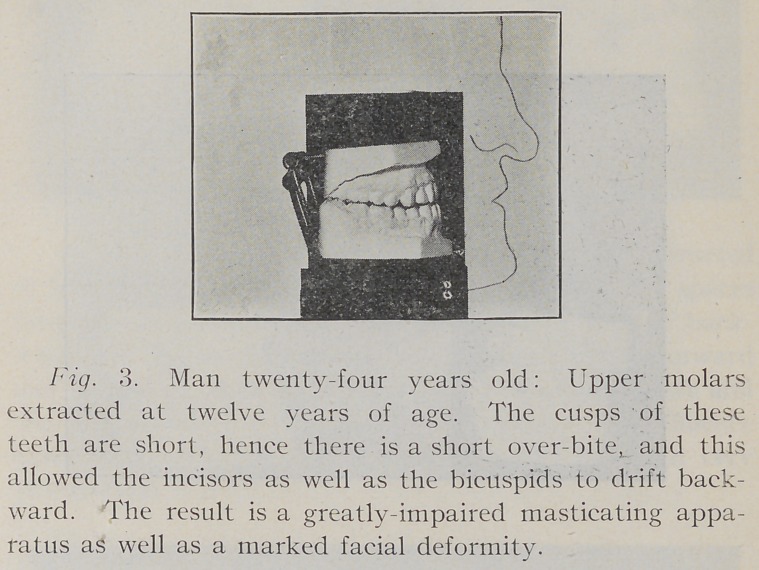


**Fig. 4. f4:**
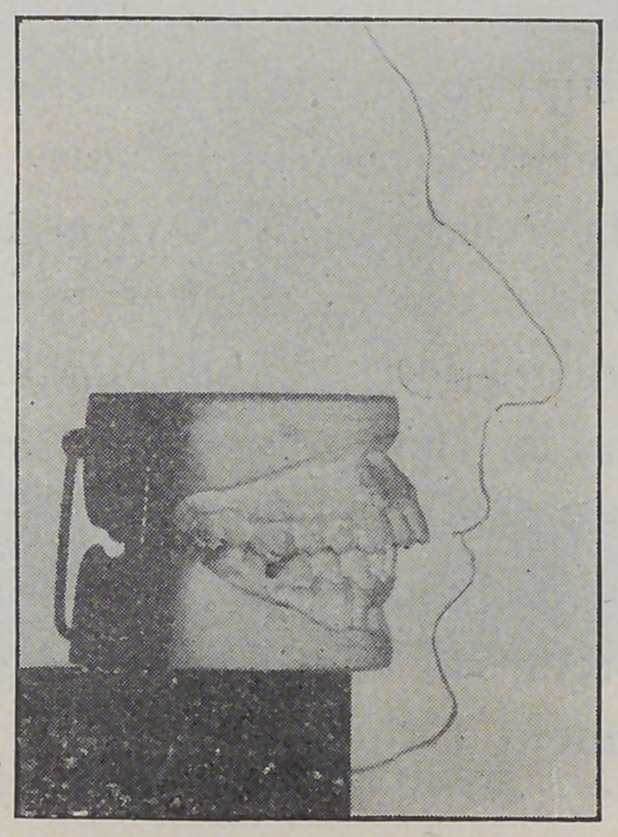


**Fig. 5. f5:**
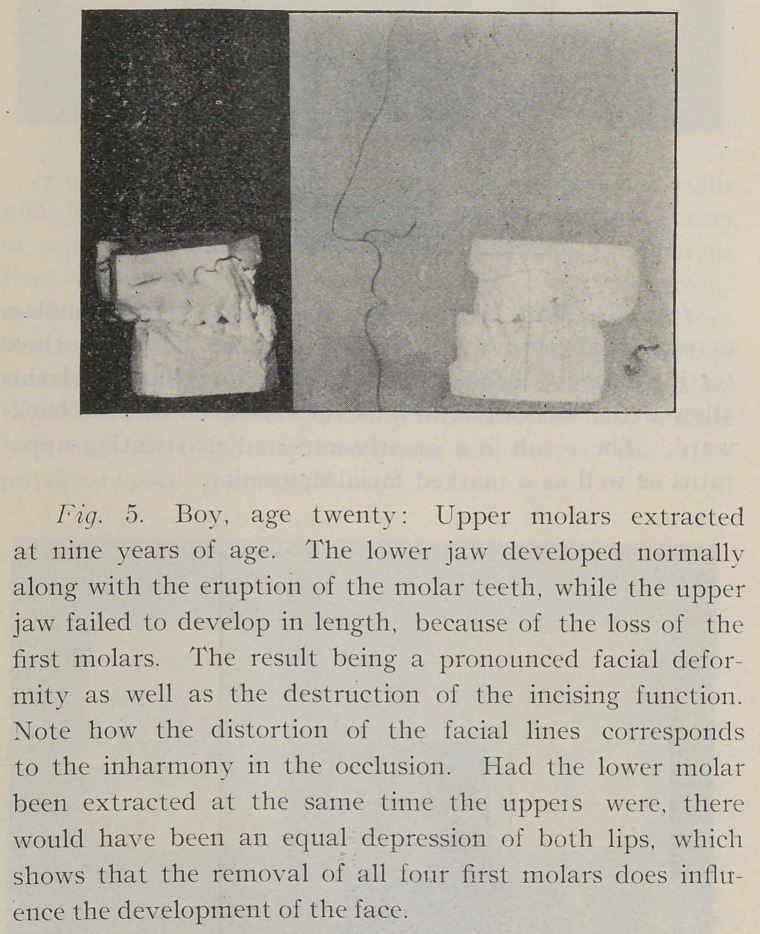


**Fig. 6. f6:**
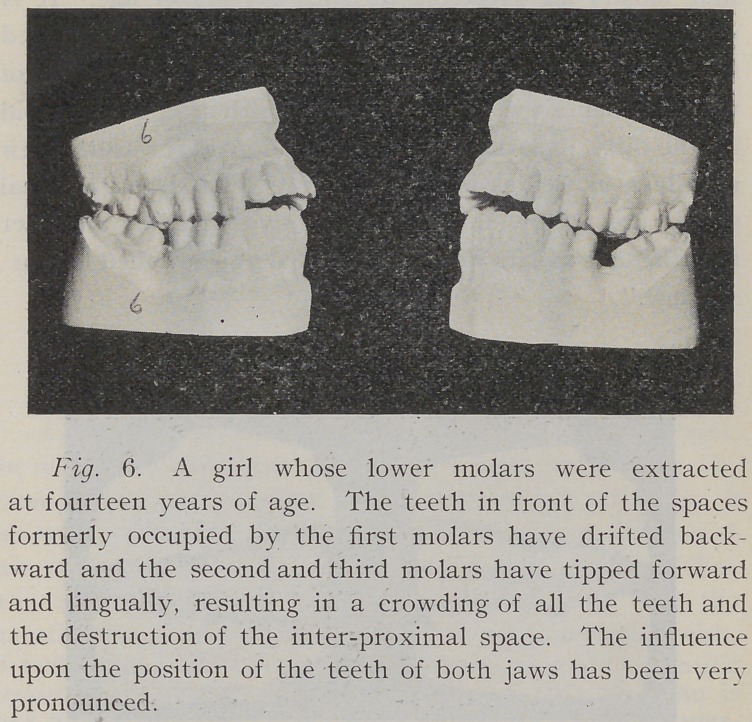


**Fig. 7. f7:**
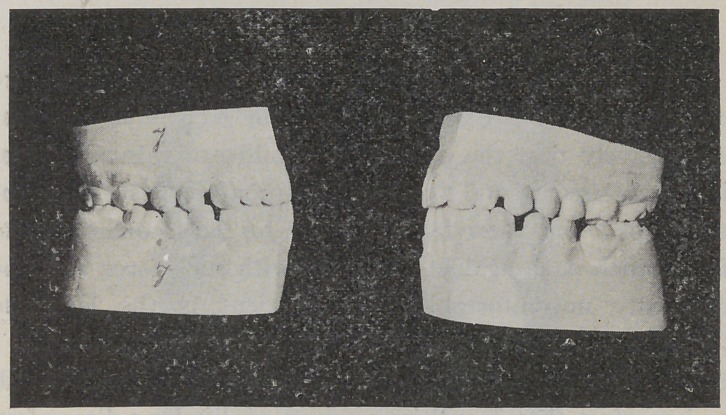


**Fig. 8. f8:**
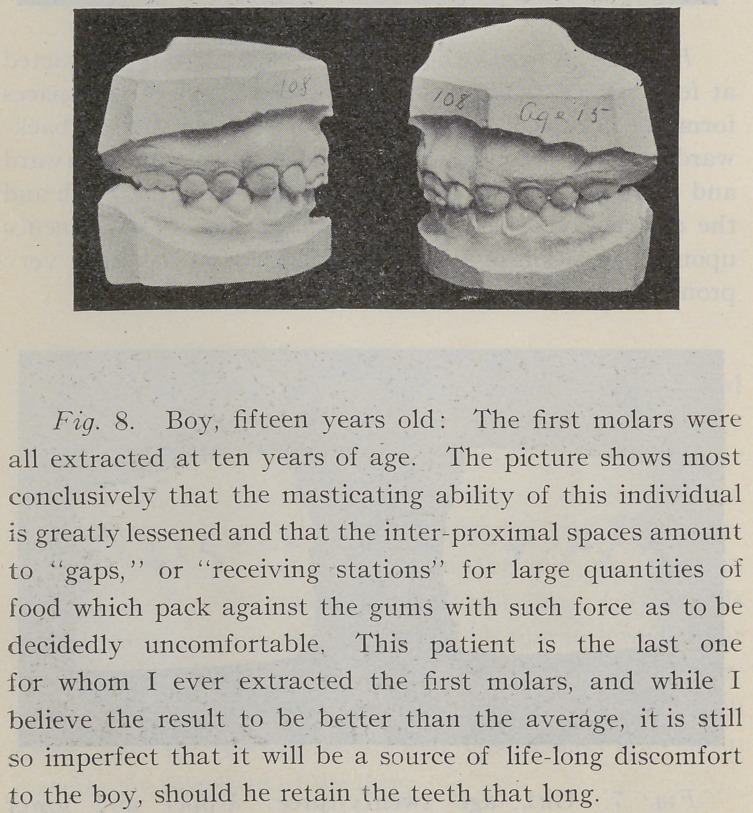


**Fig. 9. f9:**
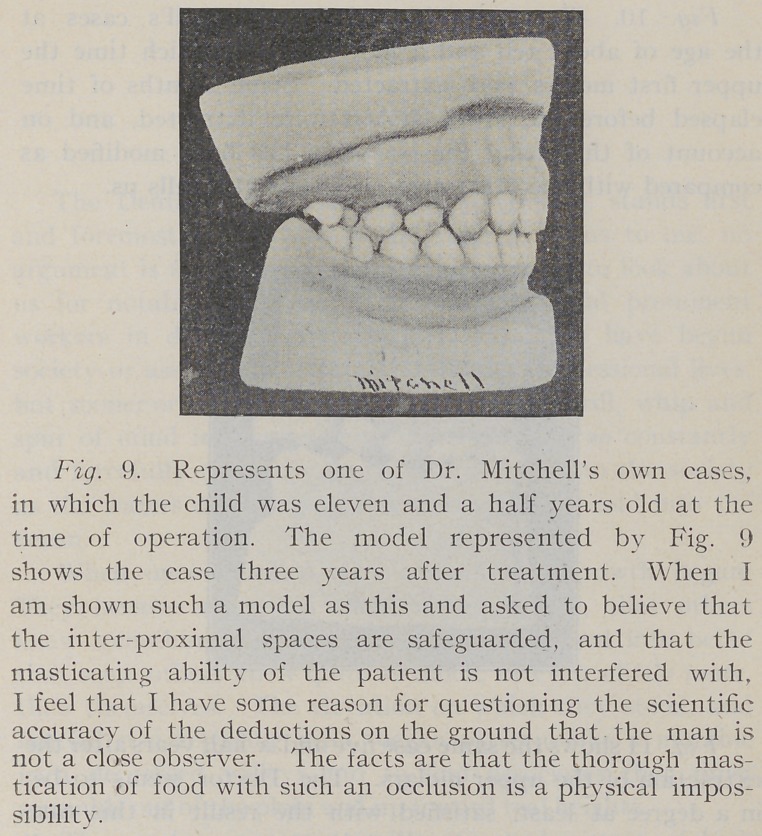


**Fig. 10. f10:**
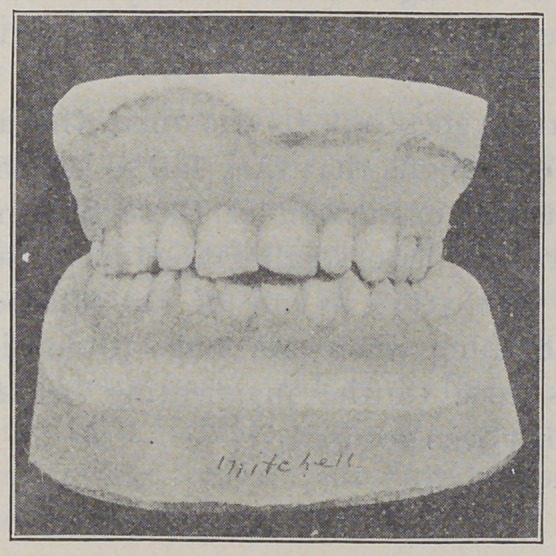


**Fig. 11. f11:**